# Supplementary material for: The Evolution and Characterization of the RNA Interference Pathways in Lophotrochozoa
Source: Genome Biol Evol. 2024 May 7;16(5):evae098. doi: 10.1093/gbe/evae098 (PMC11114477; doi:10.1093/gbe/evae098)
Supplement: evae098_Supplementary_Data [file evae098_supplementary_data.zip › Supplementary_materials.zip!/selected_miRNAs/NoGe_selected_miRNAs/mirdeep_pdf_files/NMRB01000212.1_Notospermus_geniculatus_strain_Ushimado_scaffold212,_whole_genome_shotgun_sequence_7288.pdf]

Provisional ID	:	NMRB01000212.1_Notospermus_geniculatus_strain_Ushimado_scaffold212,_whole_genome_shotgun_sequence_7288
Score total	:	930.5
Score for star read(s)	:	3.9
Score for read counts	:	921.9
Score for mfe	:	1.7
Score for randfold	:	0
Score for cons. seed	:	3
Total read count	:	1820
Mature read count	:	1813
Loop read count	:	0
Star read count	:	7

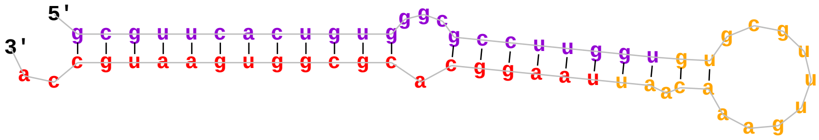

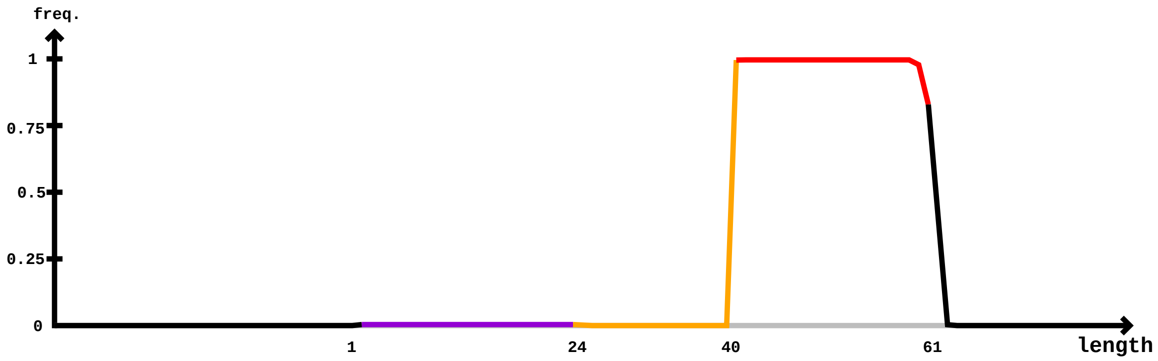


```
                                             Star                                       Mature
5'- uagcuaugccauggaaccggcucucucuaugcguucacugugggcgccuuggugugcguuugaaacaauuaaggcacgcggugaaugccaagggaaggcacuaauuucaa   -3'   obs
    uagcuaugccauggaaccggcucucucuaugcguucacugugggcgccuuggugugcguuugaaacaauuaaggcacgcggugaaugccaagggaaggcacuaauuucaa         exp
    ...........(((((...(((.(((((..((((((((((((...((((((((((.........)).)))))))).))))))))))))..))))).))).....))))).    reads     mm     sample
    .....................................................................uaaggcacgcggugaaugc......................    11        0      Mm1
    .....................................................................uaaggcacgcggugaaugcca....................    37        0      Mm1
    .....................................................................uaaggcacgcggugaaGgcca....................    1         1      Mm1

    ..............................gcguucacugugggcgccuuggu.........................................................    2         0      Mm2
    ..............................gcguucacugugggcgccuugguA........................................................    3         1      Mm2
    .....................................................................uaaggcacgcggugaaugc......................    4         0      Mm2
    .....................................................................uaaggcacgcggugaauAcc.....................    1         1      Mm2
    .....................................................................uaaggcacgcggugaaugcc.....................    21        0      Mm2
    .....................................................................uaaggcacgcggugaaCgcc.....................    1         1      Mm2
    .....................................................................uaaggcacgcggugaaugcca....................    60        0      Mm2
    .....................................................................uaaggcacgcggugaaugccG....................    3         1      Mm2
    .....................................................................uaaggcacgcggugaauUcca....................    1         1      Mm2
    .....................................................................uaaggcacgcggugaaugccU....................    8         1      Mm2
    .....................................................................uaaggcacgcggugaauAcca....................    1         1      Mm2
    .....................................................................uaaggcacgcggugaaAgcca....................    1         1      Mm2
    .....................................................................uaaggcacgcggugaaGgcca....................    1         1      Mm2
    .....................................................................uaaggcacgcggugaaugccaU...................    5         1      Mm2

    .....................................................................uaaggcacgcggugaaugcc.....................    10        0      Fm2
    .....................................................................uaaggcacgcggugaaCgcc.....................    1         1      Fm2
    .....................................................................uUaggcacgcggugaaugcca....................    1         1      Fm2
    .....................................................................uaaggcacgcggugaCugcca....................    1         1      Fm2
    .....................................................................uaaggcacgcggugaauAcca....................    2         1      Fm2
    .....................................................................uaaggcacgcAgugaaugcca....................    1         1      Fm2
    .....................................................................uaaggcacgcggugaauUcca....................    1         1      Fm2
    .....................................................................uaaggcacgcggugaaugccG....................    2         1      Fm2
    .....................................................................uaaggcacgcggugaaugcca....................    72        0      Fm2
    .....................................................................uaaggcacgcggugaaugccU....................    5         1      Fm2
    .....................................................................uaaggcacgcggugaauCcca....................    1         1      Fm2

    .....................................................................uaaggcacgcggugaaugc......................    10        0      Fm1
    .....................................................................uaaggcacgcggugaaugcc.....................    100       0      Fm1
```

```
                              Star                                  Mature
uagcuaugccauggaaccggcucucucuaugcguucacugugggcgccuuggugugcguuugaaacaauuaaggcacgcggugaaugccaagggaaggcacuaauuucaa
.....................................................................uaaggcacgcggugaaugcA.....................    1     1     Fm1
.....................................................................uaaggcacgcggugaaGgcca....................    3     1     Fm1
.....................................................................uaaggcacgcggugaaCgcca....................    1     1     Fm1
.....................................................................uaaggcacgcAgugaaugcca....................    1     1     Fm1
.....................................................................uaaggcacgcggugaauAcca....................    2     1     Fm1
.....................................................................uaaggcacgcggugaauCcca....................    5     1     Fm1
.....................................................................uaaggcacgcggugaaugcca....................    398   0     Fm1
.....................................................................uaaggcacgcggugaauUcca....................    3     1     Fm1
.....................................................................uaaggcacgcggugaaugccU....................    286   1     Fm1
.....................................................................uaaggcacgcggugaaugccG....................    27    1     Fm1
.....................................................................uaaggcacgcggugUaugcca....................    1     1     Fm1
.....................................................................uaaggcacgcggugaaugccaC...................    8     1     Fm1
.....................................................................uaaggcacgcggugaaugccaU...................    12    1     Fm1
.....................................................................uaaggcacgcggugaaugccaa...................    3     0     Fm1
.....................................................................uaaggcacgcggugaaugccaag..................    5     0     Fm1
......................................................................aaggcacgcggugaaugccU....................    1     1     Fm1

..............................gcguucacugugggcgccuuggu.........................................................    1     0     Mm3
.....................................................................uaaggcacgcggugaaugc......................    4     0     Mm3
.....................................................................uaaggcacgcggugaaCgcc.....................    1     1     Mm3
.....................................................................uaaggcacgcggugaaugcc.....................    22    0     Mm3
.....................................................................uaaggcacgcggugaaugcca....................    36    0     Mm3
.....................................................................uaaggcacgcggugaaugccU....................    6     1     Mm3
.....................................................................uaaggcacgcggugaaugccaC...................    1     1     Mm3
.....................................................................uaaggcacgcggugaaugccaU...................    2     1     Mm3
.....................................................................uaaggcacgcggugaaugccaaA..................    1     1     Mm3

..............................gcguucacugugggcgccuuggu.........................................................    1     0     Fm3
.....................................................................uaaggcacgcggugaaugc......................    4     0     Fm3
.....................................................................uaaggcacgcggugaaugcc.....................    107   0     Fm3
.....................................................................uaaggcacgcggugaauAcc.....................    3     1     Fm3
.....................................................................uaaggcacgcUgugaaugcc.....................    1     1     Fm3
.....................................................................uaaggcacgcggugaaAgcc.....................    1     1     Fm3
.....................................................................uaaggcacgcggugaauUcc.....................    1     1     Fm3
.....................................................................uaaggcacgcgUugaaugcca....................    1     1     Fm3
.....................................................................uaaggcacgcggugaaCgcca....................    2     1     Fm3
.....................................................................uaaggcacgcggugaauUcca....................    3     1     Fm3
.....................................................................uaaggcacgcggugaauAcca....................    2     1     Fm3
.....................................................................uaaggcacgcAgugaaugcca....................    1     1     Fm3
.....................................................................uaaggcacgcggugGaugcca....................    1     1     Fm3
.....................................................................uaaggcacgcggugaaGgcca....................    4     1     Fm3
.....................................................................uaaggcacgcggugaaugccU....................    62    1     Fm3
.....................................................................uaaggcacgcggugaaugcca....................    410   0     Fm3
.....................................................................uaaggcacgcggugaauCcca....................    3     1     Fm3
.....................................................................uaaggcacgcggugaaugcGa....................    1     1     Fm3
.....................................................................uaaggcacgcggugaGugcca....................    1     1     Fm3
.....................................................................uaaggcacgcggugaaAgcca....................    1     1     Fm3
.....................................................................uaaggcacgcUgugaaugcca....................    1     1     Fm3
.....................................................................uaaggcacgcggugaaugccaU...................    7     1     Fm3
.....................................................................uaaggcacgcggugaaugccaa...................    3     0     Fm3
```
